# Current pet ownership modifies the adverse association between long‐term ambient air pollution exposure and childhood asthma

**DOI:** 10.1002/clt2.12005

**Published:** 2021-03-15

**Authors:** Xiao‐Wen Zeng, Caroline J. Lodge, Adrian J. Lowe, Yuming Guo, Michael J. Abramson, Gayan Bowatte, Li‐Wen Hu, Bo‐Yi Yang, Zan‐Xiong Chen, Shyamali C. Dharmage, Guang‐Hui Dong

**Affiliations:** ^1^ Department of Occupational and Environmental Health School of Public Health Sun Yat‐sen University Guangzhou China; ^2^ Melbourne School of Population & Global Health Allergy and Lung Health Unit Centre for Epidemiology and Biostatistics The University of Melbourne Melbourne Victoria Australia; ^3^ Department of Epidemiology and Preventive Medicine School of Public Health and Preventive Medicine Monash University Melbourne Victoria Australia; ^4^ Department of Basic Sciences Faculty of Allied Health Sciences University of Peradeniya Peradeniya Sri Lanka; ^5^ National Institute of Fundamental Studies Kandy Sri Lanka; ^6^ Maternal and Child Hospital of Maoming City Maoming China

**Keywords:** air pollution, asthma, children, pet ownership

## Abstract

**Background:**

Recent studies suggest that household endotoxin and allergens can modify the impact of air pollutants on development of asthma; however, epidemiological evidence is limited and conflicting.

**Objectives:**

To investigate whether pet ownership modified the association between ambient air pollution and asthma in children.

**Methods:**

We conducted a population‐based cross‐sectional study, the Seven Northeast Cities Study in China and recruited a total of 59,754 children from 94 schools during 2012–2013. Long‐term air pollutant concentrations, including airborne particulate matter with a diameter of 1 μm or less (PM_1_), PM_2.5_, PM_10_, and nitrogen dioxide (NO_2_) from 2009 to 2012 were estimated using a random forest model. We collected information of respiratory health in children using the *Epidemiologic Standardization Project Questionnaire of the American Thoracic Society* (ATS‐DLD‐78‐A). Regression models were used to evaluate associations between pet ownership and air pollution on asthma after adjusting for potential covariates.

**Results:**

Exposure to increasing levels of air pollutants was associated with higher prevalence of asthma, but associations were significantly attenuated in children who owned pets. For example, compared to children without pets, those who owned pets did not have an increased risk of symptoms of asthma (odds ratio, 1.01, 95% confidence interval: 0.78, 1.30), wheeze (0.96, 95% confidence interval [CI]: 0.76, 1.21), and cough (1.01, 95% CI: 0.87, 1.18) for each 10 µg/m^3^ increase in PM_1_ (*P*_*‐int*_ < 0.05). Similar trends were observed for other air pollutants. Dog and bird ownership decreased the associations of asthma and cough with air pollutant exposure. The main findings were consistent with a series of sensitivity analyses.

**Conclusion:**

Current pet ownership may reduce the adverse impact of long‐term air pollution on childhood asthma. Longitudinal studies are needed to confirm this finding which could have important implications for public health.

## 1 INTRODUCTION

Asthma is the most common chronic childhood disease. It may present as wheezing, cough, and shortness of breath with varying degrees of severity.^1^ Asthma is estimated to have affected 273 million people globally in 2017, with an increase of 4.9% in age‐adjusted years lived with disability rates in the last decade.^2^ Therefore, identifying the key environmental and lifestyle factors related to asthma is important to better understand and reduce the burden of asthma among children.

Increasing evidence suggests that ambient air pollution plays a crucial role in the prevalence or incidence of childhood asthma.^3^, ^4^ There is increasing interest in understanding what factors modify the impact of air pollution on asthma and other respiratory conditions. Allergens and endotoxins are known to interact with air pollutants, demonstrating harmful,^5^ no effect,^6^ or even beneficial immunologic and clinical effects in murine models.^7^, ^8^ However, there have been few epidemiological evaluations of co‐exposure to ambient air pollution and indoor endotoxin or allergen on the prevalence of asthma.

Household pets such as cats and dogs provide a significant source of both indoor allergens and endotoxin exposure.^9^ Owning pet has become more and more popular in modern life. Exposure to indoor aeroallergens has been associated with allergic sensitization, which increases susceptibility to asthma.^10^ On the other hand, endotoxin, particularly microbiome exposure in early life has been associated with a reduced risk of asthma.^11^ While McConnell et al. reported that dog ownership may worsen associations between air pollution and respiratory symptoms in asthmatic children,^12^ Matsui et al. found clinically relevant interactions suggesting that indoor NO_2_ exposure modified the harmful impact of endotoxin on clinical outcomes in children from an asthma cohort.^13^ Both of these studies are from high‐risk groups for asthma (e.g., children with persistent asthma) and currently very limited evidence are from general pediatric population.

Given the limited epidemiological evidence assessing co‐exposure to ambient air pollution and pet ownership on childhood asthma, we used the data from Seven Northeast Cities Study (SNEC) based on a population‐based investigation with approximately 60,000 children in China to address this knowledge gap. While we have previously evaluated the association between long‐term air pollution exposure and childhood asthma,^14^ this manuscript investigated whether pet ownership modified the association between ambient air pollution exposure and asthma in children.

## 2 METHODS

### 2.1 Study population

The study sample was from SNEC, a population‐based investigation which was designed to evaluate associations of ambient air pollution and various health outcomes among children. The SNEC has been described in detail previously.^14^, ^15^, ^16^, ^17^ Briefly, all 27 urban districts in seven cities (Shenyang, Dalian, Fushun, Anshan, Benxi, Liaoyang, and Dandong city) from Liaoning province, northeastern China, were selected to maximize the intra‐ and inner‐city air pollution levels based on air monitoring data from 2009 to 2012 (Table S1). We randomly selected one or two kindergartens, elementary schools, and middle schools in each district around the area within 1–2 km from local air monitoring stations. All parents or guardians of participating children provided written informed consent and completed the study questionnaires including information of demographic characteristics, children's health and other exposure factors. From April 2012 to January 2013, a total of 68,647 children in 94 schools were recruited with the response rate of 93.1% (63,910 children). We excluded 4156 children who lived at their current address less than 2 years. Finally, 59,754 eligible children were included in this study. This study was conducted in accordance with the amended Declaration of Helsinki.

### 2.2 Ambient air pollution exposure

We estimated daily concentrations of particulate matter with an aerodynamic diameter less than 1 µm (PM_1_), 2.5 µm (PM_2.5_) or 10 µm (PM_10_), and nitrogen dioxide (NO_2_) using a random forest model at a spatial resolution of 10 × 10 km from 2009 to 2012 as previous reported.^14^ In brief, PM and NO_2_ levels were estimated by model performance using hundreds of geographic variables, including satellite remote sensing, land use, population, and meteorological data. Each participant's home address was geocoded and superimposed over the predicted air pollutant grids. Four‐year (2009–2012) daily averages of air pollutants' concentrations were calculated and assigned to each child as surrogates for long‐term air pollution exposures.^12^, ^15^ Detailed information regarding the modeling has been published elsewhere^18^, ^19^ and is presented in the eMethod 1.

### 2.3 Covariates

#### 2.3.1 Pet ownership

Data on pet ownership of the household were retrieved from the questionnaire. Children's parents or guardians were asked if the household currently owned a pet and if so, what kind of pet (dog, cat, bird, poultry, or others). Furthermore, we also collected self‐reported pet ownership during pregnancy and during the first two years of early life in childhood. We found no modification of pet ownership in utero (Table S2) and in the first two years of early life (Table S3) on the associations between air pollution exposure and childhood asthma, therefore, we focused on current pet ownership in this analysis. In this manuscript, pet ownership referred to “current pet ownership” unless otherwise specified. We examined the pet‐specific modification on the associations between air pollution exposure and asthmatic symptoms using two classifications: (1) general with one or more pet in a house; (2) exclusive one pet species in a house, such as cat only, dog only.

#### 2.3.2 Other covariates

We collected child's information (sex, age, BMI, physical exercise time, adverse birth outcome), parental information (socioeconomic status, family history of asthma), and household environment secondhand smoke (SHS) in home, per capita residential space, mold in home, household ventilation status, etc.), as reported by the parents. Potential confounders and adjusted covariates were identified a priori based on previous literature.^12^, ^20^, ^21^ A detailed description and definition of these covariates is described in the eMethod 2.

### 2.4 Outcomes

We collected information of respiratory health status in children using a widely used Epidemiologic Standardization Project Questionnaire of the American Thoracic Society (ATS‐DLD‐78‐A).^22^ The Chinese version of the ATS questionnaire was well validated and has been used in Chinese population previously.^14^, ^15^ We defined current asthma, current wheeze and current cough using positive responses to the following questions. For current asthma: “Has a doctor ever diagnosed this child having asthma”, and “Has this child experienced an asthma attack in the past two years”, or “Has this child taken drug or treatment for asthma or asthmatic bronchitis in the past two years?”. For current wheeze: “Has this child's chest ever sounded wheezy or whistling when he/she had a cold?” and “Has this child had more than two times of such episodes in the last 12 months?”. For current cough: “Has this child had cough more than four days per week and lasted for more than three months with or without an infection during the last 12 months?”

### 2.5 Statistical analysis

Air pollutant levels were treated as independent continuous or categorical predicted variables and asthmatic symptoms as dependent variables. We used a two‐level regression strategy which has been described previously to consider children at the first‐level units and study districts at the second‐level units.^14^, ^15^ The details of this model are described in the eMethod 3. We considered the interaction term of pet ownership and air pollutant levels in regression models. We did not adjust for multiple air pollutants in one model because of the inter‐correlation between air pollutants.

We further examined the robustness of the main findings with the following sensitivity analyses: (1) We categorized the air pollutant levels in tertiles and analyzed the modification of pet ownership on the associations of air pollution and asthma under different air pollution exposure levels; (2) Pet ownership during pregnancy or in the first two years of life has been associated with development or morbidity of childhood asthma. Hence, we only included children whose families never had a pet during pregnancy and in the first two years of childhood (*n* = 53,730); (3) Because previous observational studies investigated co‐exposure to pet ownership and air pollution only in asthmatic children, therefore, we analyzed a subgroup which only included asthmatic children to make our results more comparable to other studies (*n* = 4669); (4) Since atopy status has been shown to be associated with childhood asthma, we analyzed the subgroup children who had doctor‐diagnosed allergic to specific allergens (food, drug, dust, pollen, detergent, and others) (*n* = 6859); (5) Because the main purpose of this study was to investigate if a pet in home modified the effect of air pollution on asthma, we further evaluated whether other exposures (e.g., secondhand smoke) that might also interact with air pollution and pet ownership on childhood asthma. So we assessed pet × air pollution × other exposure interactions and conducted a stratified analysis if there was a significant three‐way interaction; (6) Considering the genetic predisposition of asthma, we excluded participants with family history of asthma (*n* = 4113); (7) Because adverse birth outcome was associated with deficient immune function in children, we therefore excluded participants with preterm birth or low birth weight (*n* = 7637).

All analyses were conducted using the SAS software version 9.4 (SAS Institute Inc.). The GLIMMIX procedure in SAS was used to fit the logistic mixed‐effects regression models. Statistical significance was tested using two‐tailed tests, and *p*‐values less than 0.05 or 0.1 were considered statistically significant for main effects or interactions, respectively. *p* values were not adjusted for multiple comparisons because our analyses were hypothesis driven.

## 3 RESULTS

### 3.1 Demographic characteristics and air pollution

The characteristics of the 59,754 children in the SNEC study are described in Table 1. The mean age of children was 10.3 *±* 3.6 years old (Table 1), and the asthmatic children were at younger ages compared with non‐asthmatic children (*p* < 0.001, Table S4). Among study participants, 6862 (11.5%) of them owned pets. The prevalence of current asthma (2.7% vs. 2.8%) and current wheeze (4.0% vs. 3.8%) in children without pets was similar to those with pets (*p* > 0.05), but a higher prevalence of current cough was observed in children owning pets (6.5% vs. 8.5%, *p* < 0.05). The prevalence of pet ownership ranged from 9.9% to 15.6% among studied cities (Table S1). Children who owned pets had significantly less outdoor physical exercise time than those without pets when exposed to high levels of air pollution (*p* < 0.05, Table S5). Table 2 shows the four‐year mean concentration and correlation of estimated PM_1_, PM_2.5_, PM_10_, and NO_2_ in studied regions.

**TABLE 1:** Characteristics among SNEC participants (*n* = 59,754)^a^

	Values
Covariates	
Age (year)	10.3 ± 3.6
BMI (kg/m^2^)	18.6 ± 4.5
Outdoor exercise time (hr/week)	6.6 ± 8.0
Residential area (m^2^/person)	23.6 ± 12.4
Sex	
Male	30,260 (50.6%)
Female	29,494 (49.4%)
Pet ownership	6862 (11.5%)
Annual family income (Chinese Yuan, CNY)	
≤ 9999	12,459 (20.9%)
10,000–29,999	22,170 (37.1%)
30,000–99,999	20,998 (35.1%)
≥ 100,000	4127 (6.9%)
Breastfeeding more than 3 months	39,756 (66.5%)
Low birthweight	2187 (3.7%)
Preterm birth	3217 (5.4%)
Parental education (≥high school)	43,786 (73.3%)
Secondhand smoke	27,822 (46.6%)
Mold in home	8668 (16.4%)
Home coal usage	3459 (5.8%)
Active home ventilation	34,840 (58.3%)
Family asthma history	4113 (6.9%)
Outcomes	
Current asthma	*p*_*‐*value_ = 0.784
Pet	1451 (2.7%)
No pet	192 (2.8%)
Current wheeze	*p*_*‐*value_ = 0.375
Pet	2111 (4.0%)
No pet	258 (3.8%)
Current cough	*p*_*‐*value_ < 0.001
Pet	3420 (6.5%)
No pet	584 (8.5%)

Abbreviation: SNEC, Seven Northeast Cities Study.

^a^ Values are represented as mean ± SD or as *n* (%).

**TABLE 2:** Four‐year average concentration of air pollutants (µg/m^3^) estimated in the SNEC study, 2009–2012

Air pollutants	Annual concentration (µg/m^3^)			Spearman correlation coefficient			
	Mean (SD)	Median (Q1,Q3)	WHO guidelines	PM_1_	PM_2.5_	PM_10_	NO_2_
PM_1_	46.8 (6.0)	45.2 (41.1, 52.7)	N.A	1	0.988^a^	0.973^a^	0.914^a^
PM_2.5_	54.7 (6.5)	52.4 (48.8, 60.5)	10	‐	1	0.986^a^	0.907^a^
PM_10_	97.9 (10.7)	95.6 (89.3, 107.5)	20	‐	‐	1	0.925^a^
NO_2_	34.9 (4.8)	35.5 (31.1, 39.2)	40	‐	‐	‐	1

Abbreviations: N.A, not available; NO_2_, nitrogen dioxide; PM_1_, particles with aerodynamic diameter ≤ 1.0 µm; PM_10_, particles with aerodynamic diameter ≤ 10 µm; PM_2.5_, particles with aerodynamic diameter ≤ 2.5 µm; SNEC, Seven Northeast Cities Study.

^a^ *p*_*‐*value_ < 0.05.

### 3.2 Pet ownership, air pollution, asthma

We found consistent positive associations between air pollutant exposure and asthmatic symptoms in children (*p* < 0.05, Table S6) as we previously reported.^14^ The associations of each air pollutant with asthmatic symptoms were modified by pet ownership which showed lower odds of asthmatic symptoms in children with pets than without pets (*P*_*‐int*_ < 0.05, Table 3). For example, the adjusted odds ratio (ORs) for current asthma in association with a 10 µg/m^3^ increase in PM_1_ was 1.51 (95% confidence interval [CI]: 1.33, 1.70) in children without pets, but 1.01 (95% CI: 0.78, 1.30) in children who owned pets. Similar associations were observed for current wheeze and cough, with the association between increasing air pollution exposures and asthmatic symptoms being attenuated in children with pets. For example, per 10 µg/m^3^ increase in PM_1_ (0.96 vs. 1.24), PM_2.5_ (0.97 vs. 1.23), PM_10_ (0.99 vs. 1.14), and NO_2_ (0.94 vs. 1.28), were not associated with current wheeze in children with a pet, but increased risk was observed in those without pets (*P*_*‐int*_ < 0.05).

**TABLE 3:** Adjusted ORs and 95% CIs for associations between a 10 µg/m^3^ increase in air pollution levels and asthmatic symptoms in children with current pet ownership^a^

Outcomes	No Pet (*n* = 52,892)	Pet (*n* = 6862)	*p*_*‐int*_ ^b^
Current asthma	*n* = 1451	*n* = 192	
PM_1_	1.51 (1.33, 1.70)	1.01 (0.78, 1.30)	0.004
PM_2.5_	1.49 (1.34, 1.67)	1.01 (0.80, 1.28)	0.002
PM_10_	1.29 (1.20, 1.38)	1.01 (0.87, 1.18)	0.003
NO_2_	1.63 (1.40, 1.91)	1.08 (0.77, 1.52)	0.026
Current wheeze	*n* = 2111	*n* = 258	
PM_1_	1.24 (1.12, 1.37)	0.96 (0.76, 1.21)	0.034
PM_2.5_	1.23 (1.12, 1.35)	0.97 (0.79, 1.20)	0.036
PM_10_	1.14 (1.07, 1.21)	0.99 (0.86, 1.13)	0.038
NO_2_	1.28 (1.13, 1.46)	0.94 (0.70, 1.26)	0.046
Current cough	*n* = 3420	*n* = 584	
PM_1_	1.28 (1.18, 1.38)	1.01 (0.87, 1.18)	0.004
PM_2.5_	1.25 (1.17, 1.34)	1.00 (0.87, 1.15)	0.003
PM_10_	1.14 (1.10, 1.19)	0.99 (0.91, 1.08)	0.003
NO_2_	1.35 (1.23, 1.48)	1.01 (0.83, 1.23)	0.007

^a^ Models adjusted for age, sex, BMI, parental education, family income, breastfeeding, low birth weight, preterm, residential area per person, secondhand smoke, mold in home, home coal usage, household ventilation, physical activity, family asthma history and district.

^b^ *p*_*‐int*_ in bold represented the interaction between air pollutant exposure and current pet.

Among types of household pet investigated, children owning dogs or birds had a comparatively lower prevalence of current asthma and cough associated with air pollution exposure compared to children without pets (Table 4). For example, the adjusted ORs for current asthma in children who owned dogs (OR = 1.00, 95% CI: 0.66, 1.52) or birds (OR = 0.77, 95% CI: 0.41, 1.45) with each 10 µg/m^3^ increase in PM_1_ level were significantly lower than children without pets (OR = 1.46, 95% CI: 1.29, 1.65) (*P*_*‐int*_ < 0.1). The results were similar in the analysis of one pet species only group as well (Table S7).

**TABLE 4:** Adjusted ORs and 95% CIs for associations between a 10 µg/m^3^ increase in air pollution levels and asthmatic symptoms in children with current pet ownership type^a^

	Cat (*n* = 736)	Dog (*n* = 3374)	Bird (*n* = 725)	Poultry (*n* = 482)	Other pets (*n* = 2115)
Current asthma	*n* = 19	*n* = 67	*n* = 32	*n* = 20	*n* = 52
PM_1_	1.45 (0.67, 3.14)	1.00 (0.66, 1.52)^b^	0.77 (0.41, 1.45)^b^	1.33 (0.59, 3.01)	1.03 (0.62, 1.71)
PM_2.5_	1.34 (0.67, 2.69)	1.00 (0.68, 1.47)^b^	0.75 (0.42, 1.36)^b^	1.23 (0.60, 2.53)	1.04 (0.66, 1.65)
PM_10_	1.19 (0.76, 1.85)	1.00 (0.79, 1.28)^b^	0.84 (0.59, 1.20)^b^	1.13 (0.70, 1.80)	1.06 (0.79, 1.42)
NO_2_	1.88 (0.64, 5.49)	1.12 (0.64, 1.97)	0.90 (0.41, 1.98)	1.69 (0.52, 5.47)	1.17 (0.59, 2.30)
Current wheeze	*n* = 31	*n* = 107	*n* = 24	*n* = 21	*n* = 89
PM_1_	1.72 (0.91, 3.26)	0.98 (0.70, 1.38)	0.85 (0.41, 1.80)	0.59 (0.27, 1.32)^b^	0.94 (0.63, 1.39)
PM_2.5_	1.62 (0.92, 2.88)	0.96 (0.70, 1.31)	0.84 (0.42, 1.66)	0.61 (0.29, 1.27)^b^	1.01 (0.70, 1.44)
PM_10_	1.38 (0.95, 2.01)	0.98 (0.80, 1.19)	0.85 (0.56, 1.29)	0.73 (0.45, 1.17)^b^	1.01 (0.80, 1.27)
NO_2_	2.16 (0.89, 5.24)	1.03 (0.66, 1.63)	0.83 (0.34, 1.99)	0.43 (0.15, 1.21)^b^	0.80 (0.49, 1.32)
Current cough	*n* = 72	*n* = 280	*n* = 53	*n* = 49	*n* = 164
PM_1_	0.95 (0.63, 1.44)	0.99 (0.80, 1.23)^b^	0.80 (0.49, 1.32)^b^	1.32 (0.79, 2.22)	1.01 (0.76, 1.34)
PM_2.5_	0.92 (0.63, 1.35)	0.97 (0.80, 1.19)^b^	0.80 (0.51, 1.27)^b^	1.22 (0.77, 1.93)	1.00 (0.77, 1.29)
PM_10_	0.93 (0.74, 1.19)	0.98 (0.86, 1.10)^b^	0.84 (0.63, 1.10)^b^	1.14 (0.85, 1.53)	0.99 (0.84, 1.16)
NO_2_	1.00 (0.58, 1.72)	0.97 (0.73, 1.29)^b^	0.75 (0.42, 1.36)^b^	1.58 (0.76, 3.27)	0.96 (0.66, 1.38)

*Note: n* in each outcome row indicates the number of participants with the outcome.

Abbreviations: CI, confidence interval; NO2, nitrogen dioxide; OR, odds ratio; PM1, particles with aerodynamic diameter ≤ 1.0 µm; PM10, particles with aerodynamic diameter ≤ 10 µm; PM2.5, particles with aerodynamic diameter ≤ 2.5 µm.

^a^ Models adjusted for age, sex, BMI, parental education, family income, breastfeeding, low birth weight, preterm, residential area per person, secondhand smoke, mould in home, home coal usage, household ventilation, physical activity, family asthma history and district.

^b^ represented the interaction between air pollutant exposure and pet ownership on asthmatic symptoms at *p* < 0.1.

### 3.3 Sensitivity analyses

When we categorized air pollution levels in tertiles, we found lower ORs for current asthma in children with pet with increasing air pollutant levels (Table S8). Considering pet ownership in early life could modulate childhood immune responses to allergens or air pollutants, we further examined the above associations in participants, excluding those who were exposed to pets during pregnancy and in the first two years of life. The results were consistent with the main results (Table S9). Additionally, we conducted an analysis in high‐risk subsamples of participants with ever doctor‐diagnosed asthma (35.2% of asthmatic children had current asthma, Table S10 in the supplement), and with doctor‐diagnosed allergy to a specific allergen (Table S11). Consistent and even stronger evidence was observed in these high‐risk groups, particularly in children who were allergic to allergens (*P*_*‐int*_ < 0.05). We found that the interaction of pet × air pollution only in younger children ≤12 years old (Table S12), and without indoor SHS exposure (Table S13).

In addition, we evaluated the robustness of our estimates by excluding children with family history of asthma who may have a genetic predisposition for asthma (Table S14), and with preterm or low birth weight (Table S15). The results were in line with the main findings.

## 4 DISCUSSION

To the best of our knowledge, this is the study with the largest sample size to examine potential modification by pet ownership on associations of air pollution exposure with childhood asthma. Our findings provide evidence that current pet ownership may reduce the prevalence of childhood asthma responses to long‐term air pollution exposure, particularly in the high air‐polluted areas. Specifically, dog and bird ownership seemed to attenuate the risk of asthma related to air pollution.

While the effect of ambient air pollution exposure on childhood asthma is now well established,^23^ the influence of pet ownership on the development or exacerbation of asthma is still debated. Pet ownership provides a significant source of both indoor allergens and endotoxin, which have been either positive or negative associated with asthma, depending on the timing of exposure and family history of asthma.^24^, ^25^ Additionally, pets may act as transmission vectors for outdoor environmental factors which could interact with indoor exposures.^26^ However, only a few observational studies have investigated the co‐exposure of air pollution and indoor pet ownership on asthmatic symptoms in children.

In a Baltimore study, Matsui et al.^13^ measured repeated indoor levels of airborne endotoxin, nicotine, and NO_2_, and examined the interaction between these exposures and asthma exacerbations in children. The authors found that in homes with higher NO_2_ (≥20 ppb), endotoxin levels were negatively associated with acute health care visit (hospitalization or emergency department visits for asthma) over the previous three months in children (OR = 0.80, 95% CI: 0.56, 1.14) while the opposite was observed in children living homes with lower NO_2_ (OR = 1.27, 95% CI: 0.79, 2.02, *P*_*‐int*_ = 0.05). In line with this finding, we also found a beneficial role of pet ownership on decreasing prevalence of asthma in children who were exposed to high level of air pollution. One potential explanation of our observation may due to less outdoor exercise time among children with pets compared with those without pets in high pollution areas. In our study, we observed children living in high air polluted areas who owned pets spent less exercise time outside compared to those did not have pets (Table S5 in the supplement). The less outdoor exposure time could allow children have more time to play with pets in home, thus may reduce the risk of high air pollution exposure. In addition, in the Baltimore study, among homes with no detectable nicotine in the indoor air, a high endotoxin level was associated with lower predicted probability of acute health care visit in children compared to those exposure to lower levels of endotoxin.^13^ Similarly, we also found lower risk of asthmatic symptoms associated with increasing air pollution concentration among children with pets living in homes with no SHS exposure (presumably no air nicotine levels) compared with those with pets living in homes with SHS exposure.

However, other studies reported opposite results. Using data from the Southern California Children's Health Study (CHS), Berhane et al.^27^ provided evidence that a decrease in ambient air pollution levels was significantly associated with a lower prevalence of bronchitic symptoms and the associations were stronger in asthmatic children who owned dogs compared to those without dogs (*P*_*‐int*_ < 0.05). In an asthmatic cohort from CHS, McConnell et al.^12^ suggested that dog ownership may worsen the relationship between air pollution and bronchitic symptoms, primarily in asthmatic children. A Canadian high‐risk asthma birth cohort found that exposure to dog allergen (Can f1) worsened the association between NO_2_ or SHS exposure and increased risk of incident asthma in children.^28^ The authors proposed a plausible explanation of the interaction between indoor endotoxin exposure and oxidant air pollutants on children.^12^ This observed synergistic positive association may be because simultaneous exposure to house endotoxin and air pollutants could induce reactive oxygen species and other free radicals in the lung.^29^

The mechanisms of interactions between air pollutants and allergens or endotoxins are poorly understood. A few animal models have investigated the effect of co‐exposure to air pollution and allergens in early life on asthma susceptibility in offspring. However, the results are inconsistent so far. Some animal models supported our findings that co‐exposure to air pollutants and allergens induced less allergic inflammatory response. In an asthmatic mouse model, Saravia et al.^8^ found that co‐exposure to combustion‐derived PM and home dust mite (HDM) induced a significantly lower Th2 lymphocyte level (cytokines such as IL‐4, IL‐5, IL‐10, etc.) and less Th2 mediated inflammation compared with control mice and HDM‐exposed mice. Another recent animal study suggested that ultrafine particle exposure during early life at a level close to the WHO recommended PM_2.5_ guidelines decreased inflammatory response under PM‐HDM exposure.^7^ These authors found higher IL‐10 levels, an important anti‐inflammatory cytokine secreted by regulatory T cells in mice, in the PM‐HDM group compared with the clean air‐HDM group.^7^

Another possible explanation for the observed interactions between pet and air pollutant exposure could be microbial diversity. Recent studies have highlighted a role of environmental microbial exposure on the protection against allergies and asthma in children.^30^, ^31^ It has been postulated that children's contact with farm animals and the associated high microbial exposures have reduced the risk of allergic disease.^31^ An in vitro study using human primary bronchial epithelial cells indicated that chronic exposure to microbial compounds in farm dust reduced the innate cytokine production of epithelial cells.^32^

Our results showed that pet ownership, in particular dog or bird ownership reduced the adverse association between air pollution and childhood asthmatic symptoms. In one of the few studies that examined the levels of indoor microbiota and airborne allergens, Richardson et al. found that homes occupied by dogs had significantly higher bacterial alpha diversity while cats did not have any impact on microbial diversity.^33^ Another study also suggested that the levels of airborne endotoxin were higher in homes with dogs than with cats.^34^ Mice exposed to dog‐associated house dust were protected against airway allergen challenge, exhibiting reduced Th2 cytokine production and a distinct gut microbiome composition.^35^

On the other hand, pet birds have received much less attention in epidemiological studies. A meta‐analysis of data from 11 European birth cohorts showed that ownership of birds in the first two years of early life neither increased nor decreased the risk of asthma in school‐aged children.^36^ However, the gut microbiota in bird faces have been reported to play functional roles in microbial production of short‐chain fatty acids and preventing pathogen colonization in the host.^37^ More toxicological and immunological studies are needed to investigate the interaction of air pollutants and allergens with different exposure periods.

There are some major strengths and novel features in our study. First, the sample size to the best of our knowledge, is the largest investigation among general population of children. Our population included approximately 60,000 children in 94 schools from 27 districts in Northeastern China. The previous limited studies were restricted to high risk cohorts with smaller sample sizes. Our large sample size ensured sufficient statistical power for the interaction analyses. In addition, we have collected a wide array of covariates which allowed us to comprehensively adjust for potential confounding and to assess a number of potential biases in sensitivity analyses. The effect estimates of modification of pet ownership on associations of air pollution and asthma in our study were generally strong and consistent, even after adjusting for many covariates. However, there may be also some other confounder explaining the associations that we could not evaluate. The findings may help to improve study design and reduce some of these limitations in future research.

However, we acknowledge several limitations in our study. First, the cross‐sectional design precluded concluding a causal association in SNEC. Second, the information on asthmatic symptoms and atopic status were collected from parental report questionnaires, which may have led to recall bias. We did not have data on more clinical indicators (e.g., biomarkers of airway inflammation, skin prick tests) or asthma severity and thus could not estimate asthma outcomes precisely. However, these outcomes defined using questionnaire‐based report have been widely used in previous epidemiological studies and have shown good repeatability.^14^, ^15^, ^27^ We also did not measure pet allergen concentrations in homes. Therefore, we were unable to discriminate the actual household pet allergen exposure with or without a pet in the same community, because cat and dog allergens are ubiquitous. However, endotoxin and pet allergen levels have been shown to be significantly higher in households with pets than those without.^9^ Third, the air pollutant levels were predicted using satellite‐based model with spatial resolution of 10  × 10 km which may misclassify exposure in some families. However, because concentrations of air pollutant gradually varied geographically, exposure misclassification for children who attend schools is less likely to induce large attenuation of associations.^27^ Fourth, we did not measure the chemical and biological components in the air particulate matters, such as heavy metals, pollen which may also contribute to the detrimental effects of air pollution on asthma.^38^, ^39^

Our findings suggest that current pet ownership may reduce the adverse impact of air pollutants on asthma in children. While the study design could not establish causality, these findings still highlight the importance of better understanding the interaction between indoor exposures and ambient air pollution. These environmental interactions could have important public health implications, particularly considering pet companionship is becoming increasingly common in modern society. Further studies are needed to confirm the interactions and clarify underlying biological mechanisms to improve our understanding of asthma prevention in children.

## AUTHORS' CONTRIBUTIONS

Drafting of the manuscript was by Xiao‐Wen Zeng, Caroline J. Lodge, and Shyamali C. Dharmage. Data collection were managed by Guang‐Hui Dong. Statistical analysis and data interpretation were conducted by Xiao‐Wen Zeng, Caroline J. Lodge, Adrian J. Lowe, Yuming Guo, Michael J. Abramson, Gayan Bowatte, Li‐Wen Hu, Bo‐Yi Yang, and Zan‐Xiong Chen. All authors critically revised the manuscript for important intellectual content and approved the final manuscript.

## Supporting information

Supplementary Material
