# Supplementary material for: Current pet ownership modifies the adverse association between long‐term ambient air pollution exposure and childhood asthma
Source: Clin Transl Allergy. 2021 Mar 15;11(1):e12005. doi: 10.1002/clt2.12005 (PMC8099301; doi:10.1002/clt2.12005)
Supplement: Supplementary file 1 — Supplementary Material [file CLT2-11-e12005-s001.doc]

**Supplemental Materials**

**Current pet ownership modifies the adverse association between long-term ambient air pollution exposure and childhood asthma**

**eMethod 1:** Description of Model Predicted PM_1_, PM_2.5_, PM_10_, and NO_2_ Data

**eMethod 2:** Description of Questionnaire Data Collection

**eMethod 3:** Description of the Two-level Binary Logistic Regression Model

**Table S1** The levels of air pollutants (µg/m^3^) and pet ownership among studied cities.

**Table S2** Adjusted ORs and 95% CIs for associations between a 10 µg/m^3^ increase air pollutant level and asthma in children and adolescents with *in utero* pet ownership.

**Table S3** Adjusted ORs and 95% CIs for associations between a 10 µg/m^3^ increase in increase air pollutant level and asthma in children and adolescents with pet ownership status in the first two years of life.

**Table S4** The age of children with asthmatic symptoms.

**Table S5** The physical exercise time (hours/week) among children with or without pets, by air pollution tertiles.

**Table S6** Adjusted ORs and 95% CIs for associations between a 10 µg/m^3^ increase in air pollution level and asthmatic symptoms in children (n = 59754).

**Table S7** Adjusted ORs and 95% CIs for associations between a 10 µg/m^3^ increase in air pollution level and asthmatic symptoms in children with current pet ownership type (one pet only).

**Table S8** Adjusted ORs and 95% CIs for associations between pet ownership and air pollution exposure on asthmatic symptoms in children, by air pollution tertiles.

**Table S9** Adjusted ORs and 95% CIs for associations between a 10 µg/m^3^ increase in air pollution level and asthmatic symptoms in children with pet ownership, excluding families owned pet during pregnancy and the first two years of early life (n = 6024).

**Table S10** Adjusted ORs and 95% CIs for associations between a 10 µg/m^3^ increase in air pollution level and asthmatic symptoms in asthmatic children, by current pet ownership (n = 4669).

**Table S11** Adjusted ORs and 95% CIs for associations between a 10 µg/m^3^ increase in air pollution level and asthmatic symptoms in children allergy to allergens, by current pet ownership (n = 6859).

**Table S12** Adjusted ORs and 95% CIs for associations between a 10 µg/m^3^ increase in air pollution level and asthmatic symptoms in children and adolescents with current pet ownership status, stratified by age.

**Table S13** Adjusted ORs and 95% CIs for associations between a 10 µg/m^3^ increase in air pollution level and asthmatic symptoms in children with current pet ownership status, stratified by indoor secondhand smoke (SHS).

**Table S14** Adjusted ORs and 95% CIs for associations between a 10 µg/m^3^ increase in air pollution level and asthmatic symptoms in children with current pet ownership status, excluding participants with family history of asthma (n = 4113).

**Table S15** Adjusted ORs and 95% CIs for associations between a 10 µg/m^3^ increase in air pollution level and asthmatic symptoms in children with current pet ownership status, excluding participants with low birth weight or preterm birth (n = 7637).

**eMethod 1: Description of Model Predicted PM_1_, PM_2.5_, PM_10_, and NO_2_ Data**

The prediction model for air pollutant concentrations has been published previously (Chen et al., 2018a, 2018b). Ground-monitored PM_1_, PM_2.5_ and PM_10_ were obtained from the China Atmosphere Watch Network (CAWNET) of the China Meteorological Administration (CMA). The network consisted of 96 stations across mainland China. Concentrations of PM_1_, PM_2.5_ and PM_10_ at all stations were measured with GRIMM 180 Environmental dust monitors (Model 1.108, Grimm Aerosol Technik GmbH, Ainring, Germany). Daily concentration of NO_2_ was estimated with satellite-derived OMI data (Daily Level-3 Nitrogen Dioxide Product) and other predictors. Two quality-control procedures were applied to all PM measurements: a "limit check" and "climatological check". For the limit check, we evaluated each valid PM measurement to determine whether it fell within its possible limits, otherwise, they were removed. In the climatological check, the median and standard deviation (SD) of hourly PM measurements were calculated at each PM observational site. Any PM values lying outside of more than three SDs from the median PM have been removed. Daily PM_1_, PM_2.5_, PM_10_, and NO_2_ concentrations were estimated by using a random forests model with a machine learning method matched to the children's geocoded home addresses. Briefly, each participant's home address was geocoded as a geographical longitude and latitude, and superimposed over the predicted daily PM_1_, PM_2.5_, PM_10_, and NO_2_ concentrations grids, and then the mean concentrations of air pollutants were calculated by averaging the daily concentrations for PM_1_, PM_2.5_, PM_10_, and NO_2_ over the four-year period of 2009–2012.

This random forests model is user-friendly, as there is no need to define the complex relationships between predictors (e.g., linear or nonlinear relationships and interactions). Also, the variable importance measures provided by random forests help the user to identify important variables and noise variables. The variable included in the final prediction model was listed as below:

*PM_ij_* = *AOD_ij_* + *TEMP_ij_* + *RH_ij_* + *BP_ij_* + *WS_ij_*+ *NDVI* + *Urban_cover* + *doy* + log(*elev)*

*NO_2ij_* = *OMI_ij_* + *TEMP_ij_* + *BP_ij_* + *RH_ij_* + *WS_ij_* + *NDVI_ij_*+ *Urban_cover_ij_* + *doy_i_* + *log(elev_j_)*

where *PM_2.5 ij_* is the PM_2.5_  or PM_10_ concentration on day *i* at station *j*; *NO_2ij_* is the NO_2_ concentration on day *i* at station *j*; *AOD_ij_* is the combined aerosol optical depth (AOD, merged values from Dark Blue and Dark Target); *OMI_ij_* is the satellite-derived OMI value; *TEMP*, *RH*, *BP,* and *WS* are mean temperature, relative humidity, barometric pressure, and wind speed on day i, respectively; *NDVI* is the monthly average NDVI value; *Urban_cover* is the percentage of urban cover with a buffer radius of 10 km; *doy* is day of the year; and *log(elev)* is the log transformed elevation. To evaluate the predictive ability of the final model, a 10-fold cross-validation (CV) was performed.

The results of a 10-fold cross-validation showed R^2^ values for daily and annual predictions were 55% and 75% for PM_1_, 83% and 86% for PM_2.5_, 78% and 81% for PM_10_, and 64% and 72% for NO_2_, respectively. The Root Mean Squared Error (RMSE) values for daily and annual predictions were 20.5 µg/m^3^ and 8.8 µg/m^3^ for PM_1_, 18.1 µg/m^3^ and 6.9 µg/m^3^ for PM_2.5_, 31.5 µg/m^3^ and 14.4 µg/m^3^ for PM_10_, and 12.4µg/m^3^ and 6.5 µg/m^3^ for NO_2_, respectively.

**References:**

Chen G, Knibbs L, Zhang W, et al. 2018a. Estimating spatiotemporal distribution of PM1 concentrations in China with satellite remote sensing, meteorology, and land use information. Environmental Pollution. 233, 1086-1094.

Chen G, Li S, Knibbs L, et al. 2018b. A machine learning method to estimate PM2.5 concentrations across China with remote sensing, meteorological and land use information. Science of The Total Environment. 636, 52-60.

**eMethod 2: Description of Questionnaire Data Collection**

The study questionnaire included queries on demographic characteristics of family, household environment, family diseases history, etc.

(1) Parental information: We dichotomized parent’s education as having a high school education or not, and categorized annual family income (Chinese Yuan, CNY) as: < 5000 CNY, 5000-9,999 CNY, 10,000-29,999 CNY, 30,000-99,999 CNY, or >100,000 CNY. Lower family income was defined if the annual family income was < 30,000 RMB. Family history of asthma was collected from questionnaire by the question: “Has any of the family member (grandparents, parents, parents’ siblings, etc) suffered from asthma?”.

(2) Children’s information: The BMI is defined as the body mass divided by the square of the body height (kg/m^2^). Preterm birth was defined as a gestational age < 37 weeks at delivery. Low birth weight was characterized as a birth weight < 2500 g. Breastfeeding was defined based on a positive response to question: “Has this child been breasted for more than three months?”. Physical exercise time (hours/week) was collected by question: “How long does your child spend for physical exercise outside per week?” If the duration > 5 h/week, then it was defined as active physical activity. Allergy to allergens was defined based on a positive response to question: “Has the doctor diagnosed your child allergic to any of the following items: food, medicine, dust, pollen, detergent, and others?”

(3) Household environment: Secondhand smoke (SHS) was defined based on a positive response to question: “Does anyone living with this child smoke in home?”. Mould in home was defined if there was a positive answer to question “Do the house walls get mold due to the leakage of rain or water?”. Home coal usage was defined if coal was used for cooking or heating in home. Per capita residential space (m^2^/person) was calculated by dividing the area of total living space by the number of household members. Active home ventilation was considered if the house windows open frequently in a day.

**eMethod 3:** Description of the Two-level Binary Logistic Regression Model

We assessed normality and described distributions for continuous variables as the mean ± standard deviation (SD), and categorical variables as n (%), comparing pet keeping to non-pet keeping children by Student’s *t*-test or χ^2^-test as appropriate. We considered a two-level logistic regression model in which children were the first-level units and the districts were the second-level units. At the child level, we predicted the logit of the prevalence rate for asthma by pet ownership (PO) and k covariates (X1 ….Xk) as follows:

logit [P(symptom_ij_ )] = α_j_ + λ_j_PO_ij_ + β_1_X1_ij_ + ….+ β_k_X1_ij_ + e_ij_ (1)

where the subscript j is for districts (j=1,…, 25), the subscript i is for children (i=1,..n_j_), α_j_ are the intercepts at the district level, λj are the regression coefficients for pet keeping, β_1_ …β_k_ are the regression coefficients of covariates, and e_ij_ are the random errors, assumed to have mean of zero and constant variance. The α_j_ and λ_j_ are random coefficients as they are assumed to vary across districts. In general, a district with a high α_j_ is predicted to have higher prevalence rates than a district with a low α_j_. Similarly, differences in λ_j_ indicate that the relationship between pet ownership and prevalence rates is not the same in all districts.

In districts with a high (low) λ_j_, pet ownership has a large (small) effect on prevalence rates (i.e., the difference between children with pet and children without pet is relatively large (small)). At the district level, we regressed the district-specific intercepts α_j_ and coefficients λ_j_ on the district-specific pollutant level (Z_j_) to explain the variations of α_j_ and λ_j_, as follows:

αj = α + γ1Z_j_ + u1_j_ (2)

λ_j_ = λ + γ2Z_j_ + u2_j_ (3)

Equation (2) predicts the prevalence rates in a district by Z_j_. If γ1 is positive, then adjusting for covariates, the prevalence rates are higher in districts with a higher pollutant level. Conversely, if γ_1_ is negative, then adjusting for covariates, the prevalence rates are lower in districts with a higher pollutant level. Equation (3) states that, adjusting for covariates, the relationship between prevalence rates and pet ownership in a district depends on the district’s pollutant level Z_j_. If γ2 is positive, then adjusting for covariates, the pet ownership effect on prevalence rates is larger with a higher pollutant level. Conversely, if γ2 is negative, then adjusting for covariates, the pet ownership effect on prevalence rates is smaller with a higher pollutant level. The -terms u1_j_ and u2_j_ are random errors at the district level, assumed to be independent and have mean of zero and constant variance. These random errors characterize the between-district variation and are assumed to be independent from e_ij_ at the child level. Note that α, λ, β1,…, βk, γ1, and γ2 are fixed effects and so do not vary across districts (they therefore have no subscript j to indicate district). The above models can be written as a single regression equation by substituting equations (2) and (3) into equation (1):

logit[P(symptomj)] = (α+γ1 Zj+λ BFij+β1X1ij+ ...+βkXkij+γ2Zj BFij)+(u2jBFij+u1j+ eij) (4)

The terms in the first and second parentheses in equation (4) are often called the fixed (or deterministic) and random (or stochastic) parts of the model, respectively. The product term ZjBFij is a cross-level interaction between the child-level variable BFij and the district-level variable Zj. The random error u2j BFij is different for different children, a situation that in ordinary multiple regression analysis is called heteroscedasticity.

**Table S1 The levels of air pollutants (µg/m^3^) and pet ownership among studied** cities.

| **City** | 1  Shenyang | 2  Dalian | 3  Fushun | 4  Anshan | 5  Benxi | 6  Dandong | 7  Liaoyang |
| --- | --- | --- | --- | --- | --- | --- | --- |
| PM_1_ ^a^ | 48.9+5.2 | 50.1+6.7 | 44.6+5.0 | 42.4+3.5 | 45.1+6.2 | 51.2+6.8 | 43.8+4.4 |
| PM_2.5_ ^a^ | 56.7+5.5 | 58.7+7.7 | 52.3+5.0 | 50.1+3.4 | 52.8+6.6 | 60.1+7.9 | 51.3+4.9 |
| PM_10_ ^a^ | 101.7+8.2 | 103.2+13.9 | 94.7+7.7 | 91.1+6.2 | 94.4+11.3 | 106.4+12.0 | 90.9+10.2 |
| NO_2_ ^a^ | 37.1+3.2 | 36.1+6.6 | 34.2+4.1 | 31.5+3.2 | 33.6+4.9 | 38.1+4.8 | 31.3+4.4 |
| Pet ^b^ | 1389(9.9%) | 1091(11.4%) | 1930(11.2%) | 565(12.3%) | 596(10.8%) | 662(15.6%) | 629(13.7%) |

^a^ Values were presented as mean +S.D.

^b^ Values were presented as number (percentage).

**Table S2** **Adjusted ORs and 95% CIs for associations between a 10 µg/m^3^ increase air pollutant level and asthma in children and adolescents with *in utero* pet ownership. ^a^**

^a^ Models adjusted for age, sex, BMI, parental education, family income, breastfeeding, low birth weight, preterm, per capita residential space, secondhand smoke, mould in home, home coal usage, household ventilation, physical activity, and family asthma history.

| **Outcomes** | **Pet ownership *in utero*** | | | ***P_-int_* ^b^** |
| --- | --- | --- | --- | --- |
|  | No (n= 56725) | Yes (n = 3029) | |  |
| **Current asthma** |  | |  |  |
| PM_1_ | 1.42 (1.26, 1.60) | | 1.50 (1.04, 2.16) | 0.776 |
| PM_2.5_ | 1.42 (1.27, 1.58) | | 1.45 (1.04, 2.02) | 0.890 |
| PM_10_ | 1.25 (1.16, 1.33) | | 1.26 (1.02, 1.56) | 0.929 |
| NO_2_ | 1.53 (1.31, 1.77) | | 1.82 (1.09, 3.04) | 0.516 |
| **Current wheeze** |  | |  |  |
| PM_1_ | 1.22 (1.10, 1.35) | | 1.01 (0.77, 1.34) | 0.202 |
| PM_2.5_ | 1.21 (1.11, 1.33) | | 1.02 (0.79, 1.31) | 0.181 |
| PM_10_ | 1.13 (1.07, 1.20) | | 1.01 (0.86, 1.18) | 0.168 |
| NO_2_ | 1.25 (1.11, 1.41) | | 1.08 (0.74, 1.57) | 0.452 |
| **Current cough** |  | |  |  |
| PM_1_ | 1.25 (1.16, 1.34) | | 1.10 (0.88, 1.36) | 0.253 |
| PM_2.5_ | 1.22 (1.14, 1.30) | | 1.09 (0.89, 1.33) | 0.289 |
| PM_10_ | 1.12 (1.08, 1.17) | | 1.07 (0.95, 1.22) | 0.477 |
| NO_2_ | 1.30 (1.19, 1.42) | | 1.19 (0.89, 1.59) | 0.559 |

^b^ *P_-int_* represented the interaction between air pollutant exposure and *in utero* pet ownership on asthma.

**Table S3 Adjusted ORs and 95% CIs for associations between a 10 µg/m^3^ increase in increase air pollutant level and asthma in children and adolescents with pet ownership status in the first two years of life. ^a^**

^a^ Models adjusted for age, sex, BMI, parental education, family income, breastfeeding, low birth weight, preterm, per capita residential space, secondhand smoke, mould in home, home coal usage, household ventilation, physical activity, and family asthma history.

| **Outcomes** | **Pet ownership in the first two years of life** | | | ***P_-int_* ^b^** |
| --- | --- | --- | --- | --- |
|  | No  (n = 54 690) | Yes  (n = 5 064) | |  |
| **Current asthma** |  | |  |  |
| PM_1_ | 1.42 (1.26, 1.60) | | 1.47 (1.10, 1.97) | 0.831 |
| PM_2.5_ | 1.42 (1.27, 1.58) | | 1.45 (1.11, 1.90) | 0.862 |
| PM_10_ | 1.24 (1.16, 1.33) | | 1.28 (1.08, 1.52) | 0.758 |
| NO_2_ | 1.51 (1.30, 1.76) | | 1.86 (1.24, 2.80) | 0.339 |
| **Current wheeze** |  | |  |  |
| PM_1_ | 1.22 (1.10, 1.35) | | 1.13 (0.88, 1.44) | 0.559 |
| PM_2.5_ | 1.21 (1.10, 1.33) | | 1.14 (0.91, 1.42) | 0.602 |
| PM_10_ | 1.13 (1.06, 1.20) | | 1.08 (0.94, 1.25) | 0.574 |
| NO_2_ | 1.25 (1.10, 1.41) | | 1.17 (0.85, 1.61) | 0.704 |
| **Current cough** |  | |  |  |
| PM_1_ | 1.25 (1.16, 1.35) | | 1.13 (0.95, 1.36) | 0.300 |
| PM_2.5_ | 1.22 (1.14, 1.31) | | 1.12 (0.95, 1.32) | 0.323 |
| PM_10_ | 1.13 (1.08, 1.17) | | 1.08 (0.97, 1.19) | 0.428 |
| NO_2_ | 1.31 (1.19, 1.43) | | 1.18 (0.94, 1.49) | 0d.428 |

^b^ *P_-int_* represented the interaction between air pollutant exposure and pet ownership in the first two years of life on asthma.

**Table S4 The age of children with current asthmatic symptoms. ^a^**

| Age  (year) | Current asthma | | | *P* | Current wheeze | | | *P* | Current cough | | | *P* |
| --- | --- | --- | --- | --- | --- | --- | --- | --- | --- | --- | --- | --- |
|  | Yes  (n=58111) | | No  (n=1634) |  | Yes  (n=57385) | | No  (n=2369) |  | Yes  (n=55750) | No  (n=4004) | |  |
|  | 10.4+3.6 | 8.3+3.6 | | <0.001 | 10.4+3.6 | 8.0+3.7 | | <0.001 | 10.4+3.6 | | 9.4+3.7 | <0.001 |

^a^ Values were presented as mean +S.D.

**Table S5** **The physical exercise time (hours/week) among children with or without pets, by air pollution tertiles.**

| Exposure | Physical exercise time | | |
| --- | --- | --- | --- |
|  | No pet | Pet | *P* |
| PM_1_ |  |  |  |
| Lower tertile | 5.8 ± 7.6 | 5.9 ± 8.4 | 0.069 |
| Middle tertile | 6.7 ± 7.9 | 6.9 ± 8.6 | 0.385 |
| Higher tertile | 7.0 ± 8.2 | 6.7 ± 7.9 | **0.001** |
| PM_2.5_ |  |  |  |
| Lower tertile | 5.9 ± 7.8 | 6.1 ± 8.7 | 0.105 |
| Middle tertile | 6.5 ± 7.7 | 6.6 ± 8.3 | 0.328 |
| Higher tertile | 7.1 ± 8.2 | 6.8 ± 7.9 | **0.003** |
| PM_10_ |  |  |  |
| Lower tertile | 5.9 ± 7.8 | 6.0 ± 8.7 | 0.070 |
| Middle tertile | 6.5 ± 7.7 | 6.7 ± 8.3 | 0.477 |
| Higher tertile | 7.1 ± 8.2 | 6.8 ± 7.9 | **0.003** |
| NO_2_ |  |  |  |
| Lower tertile | 6.1 ± 7.9 | 6.0 ± 8.7 | **0.001** |
| Middle tertile | 6.4 ± 7.8 | 6.7 ± 8.3 | 0.722 |
| Higher tertile | 7.1 ± 8.0 | 6.8 ± 7.9 | **0.002** |

*P* in bold represented statistically significant in outdoor exercise time among children with or without pets at *P* < 0.05.

Lower tertile (1^st^ tertile, µg/m^3^): PM_1_ < 41.3; PM_2.5_  < 49.0; PM_10_ < 90.3; NO_2_ < 31.6;

Middle tertile (2^nd^ tertile, µg/m^3^): 41.3 ≤ PM_1_ < 51.1; 49.0 ≤ PM_2.5_  < 58.7; 90.3 ≤ PM_10_ < 105.1; 31.6 ≤ NO_2_ < 38.9;

Higher tertile (3^rd^ tertile, µg/m^3^): PM_1_ ≥ 51.1; PM_2.5_  ≥ 58.7; PM_10_ ≥ 105.1; NO_2_ ≥ 38.9.

**Table S6** **Adjusted ORs and 95% CIs for associations between a 10 µg/m^3^ increase in air pollution level and asthmatic symptoms in all children (n = 59754). ^a^**

| **Outcomes** | **ORs** | **95% CI** | ***P*** |
| --- | --- | --- | --- |
| **Current asthma** (n = 1614) |  |  |  |
| PM_1_ | 1.42 | 1.27, 1.60 | < 0.001 |
| PM_2.5_ | 1.42 | 1.28, 1.57 | < 0.001 |
| PM_10_ | 1.25 | 1.17, 1.33 | < 0.001 |
| NO_2_ | 1.54 | 1.33, 1.79 | < 0.001 |
| **Current wheeze** (n = 2369) |  |  |  |
| PM_1_ | 1.20 | 1.09, 1.32 | < 0.001 |
| PM_2.5_ | 1.20 | 1.09, 1.31 | < 0.001 |
| PM_10_ | 1.12 | 1.06, 1.19 | < 0.001 |
| NO_2_ | 1.24 | 1.10, 1.39 | 0.001 |
| **Current cough** (n = 4004) |  |  |  |
| PM_1_ | 1.23 | 1.15, 1.33 | < 0.001 |
| PM_2.5_ | 1.21 | 1.13, 1.29 | < 0.001 |
| PM_10_ | 1.12 | 1.07, 1.17 | < 0.001 |
| NO_2_ | 1.29 | 1.18, 1.41 | < 0.001 |

^a^ Models adjusted for age, sex, BMI, parental education, family income, breastfeeding, low birth weight, preterm, per capita residential space, secondhand smoke, mould in home, home coal usage, household ventilation, physical activity, and family asthma history.

**Table S7** **Adjusted ORs and 95% CIs for associations between a 10 µg/m^3^ increase in air pollution level and asthmatic symptoms in children with current pet ownership type (one pet only). ^a^**

| **Outcomes** | | **No pet**  **(n = 52892)** | **Cat only**  **(n = 416)** | **Dog only**  **(n = 2737)** | **Bird only**  **(n = 489)** | **Poultry only**  **(n = 163)** | **Other pets only**  **(n = 1851)** |
| --- | --- | --- | --- | --- | --- | --- | --- |
| **Current asthma** | n = 1451 | | n = 14 | n = 54 | n = 23 | n = 11 | n = 50 |
| PM_1_ | | 1.46 (1.30, 1.66) | 1.59 (0.62, 4.06) | 0.95 (0.60, 1.52) ^*^ | 0.76 (0.35, 1.63) ^*^ | 1.65 (0.49, 5.53) | 1.08 (0.64, 1.81) |
| PM_2.5_ | | 1.46 (1.30, 1.63) | 1.46 (0.62, 3.43) | 0.99 (0.64, 1.52) ^*^ | 0.78 (0.38, 1.58) ^*^ | 1.53 (0.53, 4.43) | 1.09 (0.68, 1.76) |
| PM_10_ | | 1.27 (1.18, 1.36) | 1.28 (0.73, 2.22) | 0.99 (0.76, 1.30) ^*^ | 0.86 (0.56, 1.33) ^*^ | 1.34 (0.66, 2.71) | 1.09 (0.80, 1.48) |
| NO_2_ | | 1.58 (1.35, 1.84) | 2.32 (0.62, 8.69) | 0.95 (0.51, 1.77) | 0.89 (0.36, 2.23) | 1.58 (0.32, 7.82) | 1.22 (0.60, 2.47) |
| **Current wheeze** | n = 2111 | | n = 24 | n = 89 | n = 14 | n = 11 | n = 80 |
| PM_1_ | | 1.22 (1.10, 1.35) | 2.44 (1.10, 5.42) ^*^ | 1.08 (0.75, 1.58) | 1.14 (0.42, 3.12) | 0.58 (0.18, 1.85) | 0.94 (0.62, 1.43) |
| PM_2.5_ | | 1.21 (1.10, 1.33) | 2.26 (1.11, 4.60) ^*^ | 1.05 (0.75, 1.48) | 1.07 (0.43, 2.66) | 0.59 (0.20, 1.72) | 1.03 (0.70, 1.51) |
| PM_10_ | | 1.13 (1.06, 1.20) | 1.70 (1.06, 2.73) ^*^ | 1.03 (0.83, 1.29) | 1.01 (0.58, 1.78) | 0.75 (0.38, 1.48) | 1.02 (0.80, 1.30) |
| NO_2_ | | 1.25 (1.10, 1.42) | 3.23 (1.07, 9.73) ^*^ | 1.17 (0.71, 1.94) | 1.50 (0.40, 5.59) | 0.54 (0.13, 2.29) | 0.79 (0.46, 1.34) |
| **Current Cough** | n = 3420 | | n = 48 | n = 225 | n = 28 | n = 22 | n = 139 |
| PM_1_ | | 1.26 (1.16, 1.36) | 1.25 (0.74, 2.10) | 0.98 (0.78, 1.25) ^*^ | 0.70 (0.35, 1.38) ^*^ | 1.70 (0.73, 3.99) | 1.10 (0.80, 1.50) |
| PM_2.5_ | | 1.23 (1.15, 1.32) | 1.22 (0.75, 1.97) | 0.98 (0.78, 1.22) ^*^ | 0.72 (0.38, 1.36) ^*^ | 1.56 (0.73, 3.30) | 1.08 (0.81, 1.44) |
| PM_10_ | | 1.14 (1.09, 1.19) | 1.12 (0.82, 1.51) | 0.99 (0.86, 1.13) ^*^ | 0.84 (0.57, 1.23) | 1.35 (0.83, 2.20) | 1.05 (0.88, 1.25) |
| NO_2_ | | 1.32 (1.20, 1.45) | 1.40 (0.71, 2.76) | 0.94 (0.69, 1.28) ^*^ | 0.78 (0.35, 1.74) | 2.05 (0.65, 6.45) | 1.08 (0.72, 1.61) |

^a^ Models adjusted for age, sex, BMI, parental education, family income, breastfeeding, low birthweight, preterm, per capita residential space, secondhand smoke, mould in home, home coal usage, household ventilation, physical activity, and family asthma history.

n in each outcome row presented the number of participants with indicated outcome.

^*^ represented the interaction between air pollutant exposure and pet ownership types on asthma at *P*< 0.1.

**Table S8** **Adjusted ORs and 95% CIs for associations between pet ownership and air pollution exposure on asthmatic symptoms in children, by air pollution tertiles. ^a^**

| **Exposure** | **Current asthma** | **Current wheeze** | **Current cough** |
| --- | --- | --- | --- |
| PM_1_ |  |  |  |
| Lower tertile | 1.58 (1.12, 2.24) | 1.51 (1.13, 2.02) | 1.40 (1.16, 1.69) |
| Middle tertile | 1.34 (1.06, 1.71) | 1.12 (0.91, 1.39) | 1.47 (1.27, 1.70) |
| Higher tertile | 0.85 (0.66, 1.11) | 1.04 (0.83, 1.30) | 1.13 (0.96, 1.33) |
| *P_-int_* ^b^ | **0.007** | 0.123 | **0.052** |
| PM_2.5_ |  |  |  |
| Lower tertile | 1.55 (1.09, 2.21) | 1.54 (1.15, 2.07) | 1.67 (1.40, 1.99) |
| Middle tertile | 1.37 (1.08, 1.73) | 1.13 (0.92, 1.40) | 1.30 (1.12, 1.51) |
| Higher tertile | 0.85 (0.66, 1.10) | 1.03 (0.82, 1.28) | 1.14 (0.96, 1.34) |
| *P_-int_*^b^ | **0.007** | **0.085** | **0.006** |
| PM_10_ |  |  |  |
| Lower tertile | 1.53 (1.07, 2.18) | 1.54 (1.15, 2.06) | 1.61 (1.35, 1.92) |
| Middle tertile | 1.38 (1.09, 1.74) | 1.13 (0.92, 1.39) | 1.33 (1.15, 1.55) |
| Higher tertile | 0.85 (0.65, 1.10) | 1.02 (0.82, 1.28) | 1.13 (0.96, 1.34) |
| *P_-int_* ^b^ | **0.007** | **0.083** | **0.015** |
| NO_2_ |  |  |  |
| Lower tertile | 1.57 (1.13, 2.19) | 1.35 (1.01, 1.80) | 1.68 (1.41, 1.99) |
| Middle tertile | 1.13 (0.87, 1.46) | 1.26 (1.02, 1.56) | 1.22 (1.04, 1.43) |
| Higher tertile | 1.03 (0.80, 1.31) | 0.99 (0.79, 1.23) | 1.21 (1.04, 1.42) |
| *P_-int_* ^b^ | 0.117 | 0.152 | **0.009** |

^a^ Models adjusted for age, sex, BMI, parental education, family income, breastfeeding status, low birth weight, preterm, per capita residential space, secondhand smoke, mould in home, home coal usage, household ventilation, physical activity, and family asthma history.

^b^ *P_-int_* in bold represented the interaction between air pollutant exposure and current pet ownership on asthmatic symptoms at *P* < 0.1.

Lower tertile (1^st^ tertile, µg/m^3^): PM_1_ < 41.3; PM_2.5_ < 49.0; PM_10_ < 90.3; NO_2_ < 31.6;

Midlle tertile (2^nd^ tertile, µg/m^3^): 41.3 ≤ PM_1_ < 51.1; 49.0 ≤ PM_2.5_ < 58.7; 90.3 ≤ PM_10_ < 105.1; 31.6 ≤ NO_2_ < 38.9;

Higher tertile (3^rd^ tertile, µg/m^3^): PM_1_ ≥ 51.1; PM_2.5_  ≥ 58.7; PM_10_ ≥ 105.1; NO_2_ ≥ 38.9

**Table S9** **Adjusted ORs and 95% CIs for associations between a 10 µg/m^3^ increase in air pollution level and asthmatic symptoms in children with pet ownership, excluding families owned pets during pregnancy and the first two years of life (n = 6024). ^a^**

^a^ Models adjusted for age, sex, BMI, parental education, family income, breastfeeding, low birth weight, preterm, per capita residential space, secondhand smoke, mould in home, home coal usage, household ventilation, physical activity, and family asthma history.

| **Outcomes** | **No pet** (n = 48497) | | **Pet** (n = 5233) | ***P_-int_*  ^b^** |
| --- | --- | --- | --- | --- |
| **Current asthma** | n = 1320 | n = 119 | |  |
| PM_1_ | 1.46 (1.29, 1.66) | 0.83 (0.60, 1.14) | | **0.001** |
| PM_2.5_ | 1.45 (1.30, 1.63) | 0.85 (0.63, 1.15) | | **0.001** |
| PM_10_ | 1.27 (1.18, 1.36) | 0.90 (0.75, 1.09) | | **0.001** |
| NO_2_ | 1.55 (1.31, 1.83) | 0.84 (0.55, 1.28) | | **0.006** |
| **Current wheeze** | n = 1893 | n = 169 | |  |
| PM_1_ | 1.21 (1.08, 1.35) | 0.94 (0.71, 1.25) | | **0.095** |
| PM_2.5_ | 1.20 (1.08, 1.32) | 0.97 (0.75, 1.26) | | 0.118 |
| PM_10_ | 1.12 (1.05, 1.19) | 0.98 (0.84, 1.16) | | 0.122 |
| NO_2_ | 1.24 (1.08, 1.41) | 0.90 (0.63, 1.29) | | **0.100** |
| **Current cough** | n = 3069 | n = 410 | |  |
| PM_1_ | 1.28 (1.18, 1.38) | 1.02 (0.85, 1.22) | | **0.018** |
| PM_2.5_ | 1.25 (1.16, 1.34) | 1.00 (0.85, 1.18) | | **0.012** |
| PM_10_ | 1.14 (1.09, 1.19) | 1.00 (0.90, 1.10) | | **0.016** |
| NO_2_ | 1.33 (1.21, 1.47) | 1.03 (0.81, 1.30) | | **0.037** |

^b^ *P_-int_* in bold represented the interaction between air pollutant exposure and current pet ownership on asthmatic symptoms at *P* < 0.1.

**Table S10** **Adjusted ORs and 95% CIs for associations between a 10 µg/m^3^ increase in air pollution level and asthmatic symptoms in asthmatic children, by current pet ownership (n = 4669). ^a^**

| **Outcomes** | | **No pet** (n = 4008) | | **Pet** (n = 661) | ***P_-int_* ^b^** |
| --- | --- | --- | --- | --- | --- |
| **Current asthma** | n = 1451 | | n = 192 | |  |
| PM_1_ | | 1.01 (0.86, 1.18) | 0.69 (0.50, 0.95) | | **0.024** |
| PM_2.5_ | | 1.06 (0.92, 1.23) | 0.73 (0.54, 0.98) | | **0.016** |
| PM_10_ | | 1.04 (0.95, 1.14) | 0.84 (0.70, 1.01) | | **0.027** |
| NO_2_ | | 0.99 (0.82, 1.20) | 0.68 (0.45, 1.03) | | **0.090** |
| **Current wheeze** | n = 936 | | n = 124 | |  |
| PM_1_ | | 0.95 (0.80, 1.14) | 0.63 (0.43, 0.91) | | **0.036** |
| PM_2.5_ | | 0.97 (0.83, 1.14) | 0.66 (0.47, 0.93) | | **0.030** |
| PM_10_ | | 0.98 (0.89, 1.09) | 0.77 (0.62, 0.96) | | **0.034** |
| NO_2_ | | 0.92 (0.74, 1.15) | 0.54 (0.34, 0.87) | | **0.037** |
| **Current cough** | | n = 944 | n = 206 | |  |
| PM_1_ | | 1.19 (1.02, 1.39) | 1.04 (0.77, 1.41) | | 0.417 |
| PM_2.5_ | | 1.17 (1.02, 1.35) | 1.01 (0.77, 1.32) | | 0.300 |
| PM_10_ | | 1.10 (1.01, 1.20) | 1.00 (0.85, 1.19) | | 0.321 |
| NO_2_ | | 1.19 (0.97, 1.45) | 1.02 (0.69, 1.51) | | 0.487 |

^a^ Models adjusted for age, sex, BMI, parental education, family income, breastfeeding, low birthweight, preterm, per capita residential space, secondhand smoke, mould in home, home coal usage, household ventilation, physical activity, and family asthma history.

^b^ *P_-int_* in bold represented the interaction between air pollutant exposure and current pet ownership on asthmatic symptoms at *P* < 0d.1.

**Table S11 Adjusted ORs and 95% CIs for associations between a 10 µg/m^3^ increase in air pollution level and asthmatic symptoms in children allergy to allergens, by current pet ownership (n = 6859). ^a^**

| **Outcomes** | | **No pet** (n = 5924) | | **Pet** (n = 935) | ***P_-int_* ^b^** |
| --- | --- | --- | --- | --- | --- |
| **Current asthma** | n = 720 | | n = 91 | |  |
| PM_1_ | | 1.37 (1.14, 1.65) | 0.84 (0.56, 1.26) | | **0.022** |
| PM_2.5_ | | 1.36 (1.15, 1.61) | 0.83 (0.57, 1.21) | | **0.013** |
| PM_10_ | | 1.23 (1.11, 1.37) | 0.92 (0.72, 1.16) | | **0.018** |
| NO_2_ | | 1.56 (1.22, 1.98) | 0.97 (0.56, 1.66) | | 0.104 |
| **Current wheeze** | n = 698 | | n = 100 | |  |
| PM_1_ | | 1.30 (1.08, 1.56) | 0.79 (0.54, 1.17) | | **0.017** |
| PM_2.5_ | | 1.28 (1.09, 1.51) | 0.80 (0.56, 1.15) | | **0.013** |
| PM_10_ | | 1.18 (1.06, 1.32) | 0.87 (0.70, 1.09) | | **0.010** |
| NO_2_ | | 1.46 (1.15, 1.85) | 0.73 (0.44, 1.21) | | **0.012** |
| **Current cough** | | n = 734 | n = 134 | |  |
| PM_1_ | | 1.31 (1.12, 1.52) | 0.71 (0.51, 0.98) | | **0.001** |
| PM_2.5_ | | 1.26 (1.10, 1.44) | 0.70 (0.52, 0.95) | | **< 0.001** |
| PM_10_ | | 1.16 (1.06, 1.26) | 0.81 (0.67, 0.98) | | **0.001** |
| NO_2_ | | 1.42 (1.15, 1.74) | 0.64 (0.42, 0.98) | | **0.001** |

^a^ Models adjusted for age, sex, BMI, parental education, family income, breastfeeding, low birthweight, preterm, per capita residential space, secondhand smoke, mould in home, home coal usage, household ventilation, physical activity, and family asthma history.

^b^ *P_-int_* in bold represented the interaction between air pollutant exposure and current pet ownership on asthmatic symptoms at *P* < 0.1.

**Table S12** **Adjusted ORs and 95% CIs for associations between a 10 µg/m3 increase in air pollution level and asthmatic symptoms in children and adolescents with current pet ownership status, stratified by age. a**

| **Outcomes** | | **≤ 12 years old** | |  |  | **> 12 years old** | |  |
| --- | --- | --- | --- | --- | --- | --- | --- | --- |
|  |  | No pet  (n = 32153) | Own pet  (n = 3487) | *P_-int_* ^b^ |  | No pet  (n = 20739) | Own pet  (n = 3375) | *P_-int_* ^b^ |
| **Current asthma** | n = 1194 | | n = 132 |  |  | n = 257 | n = 60 |  |
| PM_1_ | | 1.60 (1.37, 1.86) | 1.04 (0.73, 1.48) | **0.022** |  | 1.52 (1.18, 1.95) | 1.05 (0.65, 1.71) | 0.156 |
| PM_2.5_ | | 1.59 (1.39, 1.82) | 1.06 (0.77, 1.45) | **0.015** |  | 1.54 (1.21, 1.96) | 1.04 (0.65, 1.66) | 0.117 |
| PM_10_ | | 1.35 (1.23, 1.47) | 1.05 (0.85, 1.28) | **0.019** |  | 1.28 (1.10, 1.49) | 1.02 (0.76, 1.36) | 0.132 |
| NO_2_ | | 1.63 (1.35, 1.98) | 1.08 (0.68, 1.70) | **0.091** |  | 1.62 (1.17, 2.26) | 1.18 (0.62, 2.25) | 0.362 |
| **Current wheeze** | n = 1745 | | n = 169 |  |  | n = 366 | n = 89 |  |
| PM_1_ | | 1.48 (1.29, 1.69) | 1.30 (0.94, 1.79) | 0.437 |  | 1.34 (1.09, 1.64) | 1.17 (0.79, 1.74) | 0.528 |
| PM_2.5_ | | 1.45 (1.29, 1.64) | 1.30 (0.97, 1.73) | 0.433 |  | 1.35 (1.11, 1.64) | 1.16 (0.79, 1.69) | 0.459 |
| PM_10_ | | 1.26 (1.16, 1.36) | 1.17 (0.97, 1.41) | 0.468 |  | 1.21 (1.07, 1.36) | 1.10 (0.87, 1.39) | 0.468 |
| NO_2_ | | 1.35 (1.16, 1.59) | 1.14 (0.75, 1.72) | 0.420 |  | 1.47 (1.13, 1.92) | 1.29 (0.76, 2.19) | 0.656 |
| **Current cough** | n = 2446 | | n = 336 |  |  | n = 974 | n = 248 |  |
| PM_1_ | | 1.35 (1.22, 1.50) | 1.02 (0.81, 1.28) | **0.020** |  | 1.35 (1.18, 1.56) | 1.20 (0.93, 1.55) | 0.365 |
| PM_2.5_ | | 1.31 (1.20, 1.44) | 1.01 (0.83, 1.24) | **0.016** |  | 1.35 (1.18, 1.55) | 1.17 (0.92, 1.50) | 0.280 |
| PM_10_ | | 1.18 (1.11, 1.25) | 1.00 (0.88, 1.14) | **0.018** |  | 1.19 (1.10, 1.30) | 1.10 (0.95, 1.28) | 0.295 |
| NO_2_ | | 1.34 (1.18, 1.52) | 0.94 (0.70, 1.25) | **0.020** |  | 1.49 (1.25, 1.77) | 1.34 (0.96, 1.86) | 0.547 |

^a^ Models were adjusted for sex, BMI, parental education, family income, breastfeeding, low birthweight, preterm, per capita residential space, secondhand smoke, mould in home, home coal usage, household ventilation, physical activity, and family asthma history.

^b^ *P_-int_* in bold represented the interaction between air pollutant exposure and pet ownership on asthmatic symptoms at *P* < 0.1.

**Table S13 Adjusted ORs and 95% CIs for associations between a 10 µg/m^3^ increase in air pollution level and asthmatic symptoms in children with current pet ownership status, stratified by indoor secondhand smoke (SHS). ^a^**

| **Outcomes** | | **No SHS exposure** | |  | **SHS exposure** | |  |
| --- | --- | --- | --- | --- | --- | --- | --- |
|  |  | No pet  (n = 28931) | Own pet  (n = 3001) | *P_-int_* ^b^ | No pet  (n = 23961) | Own pet  (n = 3861) | *P_-int_* ^b^ |
| **Current asthma** | n = 717 | | n = 80 |  | n = 734 | n = 112 |  |
| PM_1_ | | 1.49 (1.25, 1.74) | 0.84 (0.56, 1.25) | **0.007** | 1.50 (1.27, 1.77) | 1.13 (0.80, 1.58) | 0.117 |
| PM_2.5_ | | 1.46 (1.26, 1.69) | 0.85 (0.58, 1.22) | **0.006** | 1.49 (1.28, 1.73) | 1.12 (0.82, 1.53) | **0.087** |
| PM_10_ | | 1.28 (1.16, 1.40) | 0.88 (0.69, 1.10) | **0.002** | 1.28 (1.17, 1.41) | 1.11 (0.91, 1.35) | 0.167 |
| NO_2_ | | 1.67 (1.34, 2.08) | 0.79 (0.47, 1.30) | **0.006** | 1.60 (1.29, 1.99) | 1.38 (0.87, 2.20) | 0.557 |
| **Current wheeze** | n = 1 066 | | n = 98 |  | n = 1 045 | n = 160 |  |
| PM_1_ | | 1.22 (1.06, 1.40) | 0.92 (0.64, 1.33) | 0.155 | 1.21 (1.05, 1.40) | 0.95 (0.71, 1.27) | 0.115 |
| PM_2.5_ | | 1.20 (1.05, 1.36) | 0.93 (0.67, 1.31) | 0.163 | 1.21 (1.06, 1.38) | 0.97 (0.74, 1.26) | 0.114 |
| PM_10_ | | 1.12 (1.04, 1.22) | 0.95 (0.77, 1.18) | 0.132 | 1.13 (1.04, 1.22) | 0.99 (0.84, 1.17) | 0.142 |
| NO_2_ | | 1.29 (1.08, 1.54) | 0.95 (0.59, 1.52) | 0.225 | 1.25 (1.05, 1.49) | 0.90 (0.62, 1.32) | 0.112 |
| **Current cough** | n = 1 542 | | n = 222 |  | n = 1 878 | n = 362 |  |
| PM_1_ | | 1.29 (1.16, 1.44) | 0.91 (0.72, 1.16) | **0.007** | 1.25 (1.13, 1.38) | 1.07 (0.88, 1.31) | 0.156 |
| PM_2.5_ | | 1.25 (1.13, 1.38) | 0.90 (0.72, 1.12) | **0.005** | 1.23 (1.12, 1.35) | 1.06 (0.88, 1.26) | 0.113 |
| PM_10_ | | 1.14 (1.07, 1.22) | 0.93 (0.81, 1.08) | **0.007** | 1.13 (1.07, 1.20) | 1.03 (0.92, 1.15) | 0.108 |
| NO_2_ | | 1.37 (1.19, 1.57) | 0.93 (0.68, 1.27) | **0.022** | 1.30 (1.15, 1.48) | 1.05 (0.81, 1.35) | 0.117 |

^a^ Models were adjusted for age, sex, BMI, parental education, family income, breastfeeding, low birthweight, preterm, per capita residential space, mould in home, home coal usage, household ventilation, physical activity, and family asthma history.

^b^ *P_-int_* in bold represented the interaction between air pollutant exposure and pet ownership on asthmatic symptoms at *P* < 0.1.

**Table S14** **Adjusted ORs and 95% CIs for associations between a 10 µg/m^3^ increase in air pollution level and asthmatic symptoms in children with current pet ownership status, excluding participants with family history of asthma (n = 4113). ^a^**

| **Outcomes** | | **No pet (n = 49392)** | | **Pet (n = 6249)** | ***P_-int_* ^b^** |
| --- | --- | --- | --- | --- | --- |
| **Current asthma** | n = 1202 | | n = 149 | |  |
| PM_1_ | | 1.50 (1.33, 1.71) | 0.88 (0.66, 1.18) | | **0.001** |
| PM_2.5_ | | 1.48 (1.32, 1.66) | 0.90 (0.69, 1.18) | | **0.001** |
| PM_10_ | | 1.28 (1.19, 1.38) | 0.94 (0.80, 1.12) | | **0.001** |
| NO_2_ | | 1.64 (1.39, 1.94) | 0.88 (0.60, 1.29) | | **0.002** |
| **Current wheeze** | n = 1840 | | n = 218 | |  |
| PM_1_ | | 1.23 (1.10, 1.37) | 0.94 (0.73, 1.20) | | **0.039** |
| PM_2.5_ | | 1.22 (1.10, 1.34) | 0.96 (0.77, 1.21) | | **0.053** |
| PM_10_ | | 1.13 (1.06, 1.21) | 0.97 (0.84, 1.13) | | **0.048** |
| NO_2_ | | 1.28 (1.12, 1.46) | 0.87 (0.63, 1.20) | | **0.024** |
| **Current cough** | | n = 3035 | n = 503 | |  |
| PM_1_ | | 1.32 (1.22, 1.43) | 1.00 (0.85, 1.18) | | **0.002** |
| PM_2.5_ | | 1.28 (1.19, 1.38) | 0.99 (0.85, 1.15) | | **0.002** |
| PM_10_ | | 1.16 (1.11, 1.21) | 0.99 (0.90, 1.09) | | **0.003** |
| NO_2_ | | 1.41 (1.27, 1.55) | 1.01 (0.82, 1.25) | | **0.004** |

^a^ Participants with family history of asthma were excluded (n = 4113). Models adjusted for age, sex, BMI, parental education, family income, breastfeeding, low birth weight, preterm, per capita residential space, secondhand smoke, mould in home, home coal usage, household ventilation, and physical activity.

^b^ *P_-int_* in bold represented the interaction between air pollutant exposure and pet ownership on asthmatic symptoms at *P* < 0.1.

**Table S15** **Adjusted ORs and 95% CIs for associations between a 10 µg/m^3^ increase in air pollution level and asthmatic symptoms in children with current pet ownership status, excluding participants with low birth weight or preterm birth (n = 7637). ^a^**

| **Outcomes** | | **No pet (n = 46371)** | | **Pet (n = 5746)** | ***P_-int_* ^b^** |
| --- | --- | --- | --- | --- | --- |
| **Current asthma** | n = 1174 | | n = 136 | |  |
| PM_1_ | | 1.48 (1.30, 1.68) | 0.88 (0.65, 1.19) | | **0.002** |
| PM_2.5_ | | 1.46 (1.30, 1.65) | 0.90 (0.68, 1.20) | | **0.001** |
| PM_10_ | | 1.28 (1.18, 1.38) | 0.94 (0.79, 1.13) | | **0.002** |
| NO_2_ | | 1.60 (1.35, 1.89) | 0.92 (0.61, 1.37) | | **0.010** |
| **Current wheeze** | n = 1768 | | n = 203 | |  |
| PM_1_ | | 1.21 (1.09, 1.36) | 0.99 (0.76, 1.28) | | 0.126 |
| PM_2.5_ | | 1.20 (1.09, 1.33) | 1.00 (0.79, 1.27) | | 0.138 |
| PM_10_ | | 1.12 (1.05, 1.20) | 1.00 (0.86, 1.17) | | 0.153 |
| NO_2_ | | 1.25 (1.09, 1.43) | 0.95 (0.68, 1.33) | | 0.125 |
| **Current cough** | | n = 2827 | n = 457 | |  |
| PM_1_ | | 1.31 (1.20, 1.42) | 1.02 (0.86, 1.21) | | **0.007** |
| PM_2.5_ | | 1.27 (1.18, 1.37) | 1.00 (0.85, 1.17) | | **0.005** |
| PM_10_ | | 1.15 (1.10, 1.21) | 0.99 (0.90, 1.09) | | **0.004** |
| NO_2_ | | 1.38 (1.24,1.53) | 1.02 (0.81, 1.27) | | **0.012** |

^a^ Participants with preterm and low birth weight were excluded (n = 7637). Models adjusted for age, sex, BMI, parental education, family income, breastfeeding, per capita residential space, secondhand smoke, mould in home, home coal usage, household ventilation, physical activity, and family asthma history.

^b^ *P_-int_* in bold represented the interaction between air pollutant exposure and pet ownership on asthmatic symptoms at *P* < 0.1.
